# Elucidating the Complex Structural and Molecular Mechanisms Driving P-Glycoprotein-Mediated Transport of Cardiac Glycosides

**DOI:** 10.3390/ijms26167813

**Published:** 2025-08-13

**Authors:** Rohit Katti, Amanda M. Kozarich, Gershon A. K. Mensah, Michael G. Bartlett

**Affiliations:** 1 Department of Pharmaceutical and Biomedical Science, University of Georgia, Athens, GA 30602, USA; rohit.katti@uga.edu (R.K.); amanda.kozarich@uga.edu (A.M.K.); 2 Complex Carbohydrate Research Center, University of Georgia, Athens, GA 30602, USA; gershon.akesemensah@uga.edu

**Keywords:** P-glycoprotein, lipid bilayer, cardiac glycosides, digoxin, Pgp-mediated transport, nuclear magnetic resonance

## Abstract

P-glycoprotein (Pgp) plays a significant role in the disposition of cardiac glycoside (CG) drugs across the cell membrane. The relatively narrow therapeutic indices of these drugs, coupled with the co-administration of drugs that inhibit Pgp’s transport mechanism, often cause an increased level of CG in the patient’s plasma, resulting in fatal arrhythmia. Therefore, understanding the underlying mechanism of the CG–Pgp interaction is necessary to circumvent Pgp-mediated transport and effectively design next-generation CGs. In this study, we conducted a comparative analysis to examine the interaction with Pgp and further understand the Pgp-mediated transport of digoxin, digitoxin, digoxigenin, and digitoxigenin. Through the drug-induced kinetic studies of Pgp, our findings suggest that each of the four drugs tested has a single binding site within Pgp. The CG–Pgp binding studies demonstrated that digoxin, digitoxin, and digoxigenin had relatively higher binding affinities. The CG-mediated conformational changes in Pgp indicated that each of the drugs shifts Pgp to an “outward-open” conformation in a nucleotide-dependent manner. STDD NMR indicated that the protons within the δ-lactone ring and the tri-D-digitoxose sugar moieties (glycones) predominantly interact with Pgp. Finally, a model was proposed for CG-induced Pgp-mediated ATP hydrolysis and transport by integrating our data with previously published Pgp-mediated CG transport results.

## 1. Introduction

P-glycoprotein (Pgp), also known as multidrug resistance protein-1 (MDR1), is a member of the ATP-binding cassette (ABC) superfamily and plays a pivotal role in the disposition of a range of drugs, including chemotherapeutics, cardiovascular drugs, and neurotherapeutics across the plasma membrane through ATP hydrolysis [1,2,3]. Pgp is localized in the apical membranes of cells that comprise excretory tissues and physiological barriers, such as the kidneys, liver, blood-brain barrier, and blood-testis barrier [4,5]. The structure of Pgp is highly flexible and consists of two homologous transmembrane domains (TMDs) [6]. Each TMD comprises six hydrophobic α-helices located in the membrane and one hydrophilic nucleotide-binding domain (NBD) located in the cytoplasm [7]. X-ray crystallography of Pgp shows an internal cavity of 6000 Å^3^ located in the cytosol, which contains several binding sites involved in the formation of hydrogen bonds and van der Waals interactions with a range of substrates, making Pgp a promiscuous protein molecule [7,8].

Over the past three decades, Pgp has sparked interest in the scientific community due to its role in the treatment of cardiovascular disease, primarily because of the narrow therapeutic indices of these drugs and their interaction with Pgp [9,10]. Due to the narrow therapeutic indices of several cardiovascular drugs, they are often co-administered with Pgp inhibitors, resulting in increased bioavailability and decreased clearance, leading to Pgp-mediated adverse drug reactions (ADRs) and sometimes causing death [10,11].

Cardiac glycosides (CGs) are a class of cardiovascular drugs derived from *Digitalis lanata*, commonly known as the foxglove plant. They are used to treat mild to moderate arrhythmia and atrial fibrillation [12]. CGs target Na^+^/K^+^ ATPase, a membrane-bound ATP-dependent enzyme, by blocking its ability to transport sodium ions across the plasma membrane [13,14]. The change in sodium ion gradient directly affects the movement of calcium ions from the cytosol to the extracellular space, resulting in a significant increase in calcium ion uptake by the cardiac sarcoplasmic reticulum, which induces a positive inotropic effect and thereby increases cardiac contractility [15,16]. Na^+^/K^+^ ATPase exhibits E1 and E2 conformational states, and CGs bind to the E2-P ground state of Na^+^/K^+^ ATPase enzyme extracellularly [17]. Previous studies have demonstrated that the presence of sugar groups on CGs influences the interaction between the drug and the enzyme, and increasing the number of hydroxyl groups in the steroid core decreases the inhibition of Na^+^/K^+^ ATPase [18].

**Figure 1:**
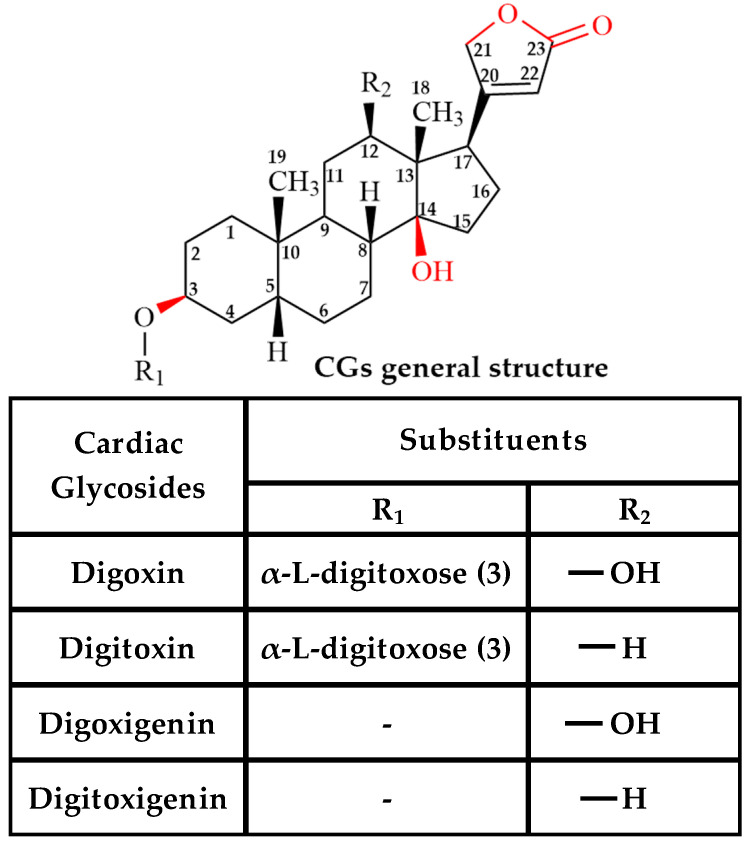
The basic structure of cardiac glycoside (CG) analogs. The table shows the difference between digoxin, digitoxin, digoxigenin, and digitoxigenin.

The plant genus *Digitalis* (foxglove) has been well known for its therapeutic value since the 18th century, and over a hundred cardenolide drugs have been identified and isolated from these plants since then [13]. Based on the presence or absence of sugar groups, CGs can be broadly classified into glycones and aglycones [19]. The general structure of CGs includes a steroid core with a lactone ring at the C17 position (Figure 1) and sugar groups, such as fucose, digitalose, digitoxose, and glucose, attached to the steroid ring at the C3 position [13,20]. Digoxin and digitoxin are the most well-known glycones of CGs used in the treatment of congestive heart failure, and they are known to be highly toxic when prescribed at high doses [21]. The aglycones of digitoxin and digoxin are digoxigenin and digitoxigenin, respectively, and they are also used in the treatment of congestive heart failure [22,23]. Previous studies using patient tumor samples and human cancer cell lines demonstrated that CGs, such as digoxin, ouabain, digitoxin, and digitoxigenin, exhibit apoptotic and antiproliferative effects on breast [24], lung [25], prostate [26,27], and renal cancer cells [24,28]. Although several preliminary studies have been conducted on the effects of CGs on tumor cells, the exact mechanism underlying these effects remains unclear.

Although CGs share the same core structure, they differ significantly in their pharmacological activity. The steroid core is believed to be a pharmacophore, responsible for binding to the receptor [29]. The two oxygen atoms on the lactone ring create a higher negative potential, resulting in increased hydrogen bonding to a five-membered unsaturated butyrolactone ring [22,30]. Finally, the sugar groups present on the glycones affects the solubility, binding, permeability, activity, and toxicity of CGs [22,29]. CGs, such as digoxin and digitoxin, are predominantly transported by the efflux pump, Pgp [31,32]. They are primarily absorbed in the small intestine and excreted via renal clearance [33]. In a 2012 study, a series of Pgp mutants were generated, and it was observed that the digoxin–Pgp interaction was decreased for mutants F336A and I340A by up to two- to four-fold when compared to wild-type Pgp, and it was also found that the lactone ring and the sugar group attached to the steroid core influenced the CG–Pgp interaction [32]. Previous studies have shown that the inhibition of Pgp in the kidneys by inhibitors such as verapamil and amiodarone increases the digoxin concentration in patient plasma, leading to digoxin toxicity [34,35,36]. In vitro studies using Pgp-expressing Caco-2 and LLC-PK1 cells transfected with human MDR1 suggested that the transport rate of digitoxin was similar to that of digoxin and decreased significantly upon the addition of 1 µM PSC-833, an analog of cyclosporin, a Pgp inhibitor [31]. In CLEFF9 cells, a Caco-2 subclone with high Pgp expression, treatment with erythromycin did not affect the Pgp-mediated efflux rate of digoxin. However, the transport rate of digoxigenin decreased by about 43% [37]. A previous study on the intracellular accumulation of digitoxigenin in MDCK cell lines overexpressing Pgp suggested no significant difference in the accumulation of the drug in cells [38]. Although the Pgp-mediated transport of digitalis-like compounds, such as digoxigenin and digitoxigenin, is documented, the underlying mechanism of their interaction with Pgp is not well understood. The inclusion of these drugs in this study is important due to their structural similarity to digoxin and its derivatives. Collectively, these studies suggest the order of efflux rates for these CGs as digitoxin > digoxin > digoxigenin > digitoxigenin. However, the type of cell line, passage number, and method used to determine the transport rate make it challenging for a detailed comparison and analysis of the results. Additionally, the underlying mechanism of the Pgp–CG interaction remains poorly understood.

This study aims to elucidate the underlying mechanism, identifying the functional groups of CGs that interact with Pgp. Identifying the functional groups that interact with Pgp and potential modification of the drug could lead to better design and development of next-generation CGs that have lower affinity for the transporter, thereby enabling them to circumvent Pgp-mediated efflux effectively. We also examined the structural and molecular mechanisms that influence Pgp-mediated efflux of CGs, as it is crucial to understand the underlying mechanism of Pgp-mediated transport to effectively design drugs that can successfully evade the toxicity caused by adverse drug reactions (ADRs). To achieve this objective, we investigated the interplay between the drug and ATP by measuring Pgp-mediated ATP hydrolysis in the presence of a range of concentrations of digoxin, digitoxin, digoxigenin, and digitoxigenin. We investigated the drug-induced conformational changes in Pgp using fluorescence spectroscopy to understand the structural mechanisms underlying Pgp-mediated efflux of CG drugs. To investigate the binding affinity of CGs to Pgp, we examined it by probing the quenching of intrinsic tryptophan residues of Pgp by the drugs. The interaction of specific functional groups on drugs with Pgp was examined using saturation transfer double-difference (STDD) NMR. Finally, a model for CG-induced Pgp-mediated ATP hydrolysis and transport was proposed by combining our results and previously published results on Pgp-mediated transport of CGs.

## 2. Results

### 2.1. Effect of Cardiac Glycosides (CGs) on Pgp-Mediated ATP Hydrolysis

The effect of CGs on Pgp-mediated ATP hydrolysis was determined using an ATPase activity assay. Pgp-mediated transport of substrates is an energy-dependent process that hydrolyses ATP, releasing inorganic phosphate (*P_i_*) [39]. Previously, it has been demonstrated that, to complete one catalytic cycle of Pgp, at least one molecule of ATP must be hydrolyzed [40,41]. Over several decades, drug-induced Pgp-mediated ATP hydrolysis has been used to identify Pgp substrates and inhibitors [42,43].

**Figure 2:**
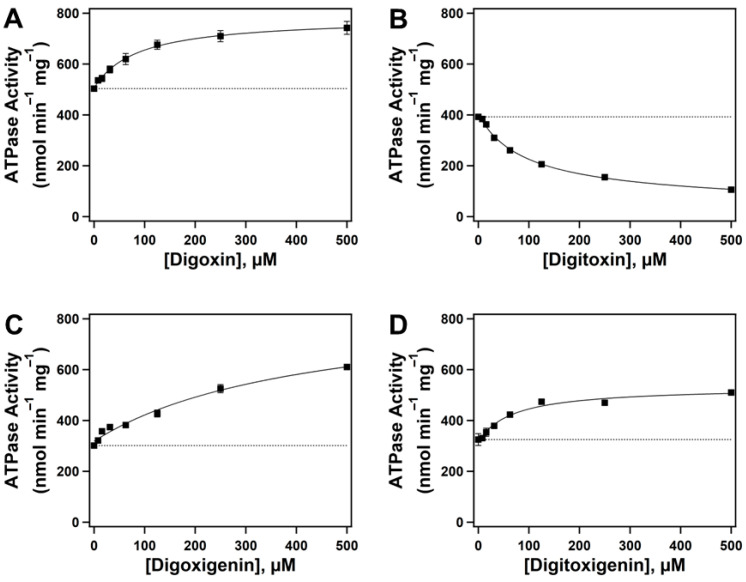
The effect of cardiac glycosides (CGs) on Pgp-mediated ATP hydrolysis. Drug-induced ATPase activity of Pgp in the presence of increasing concentrations of (**A**) digoxin (closed squares), (**B**) digitoxin (closed squares), (**C**) digoxigenin (closed squares), and (**D**) digitoxigenin (closed squares). The dotted line in each panel represents the basal activity in the absence of the drug. Each data point represents the average of at least three replicates. Error bars indicate the standard deviation.

Figure 2 demonstrates the effect of CGs on Pgp-mediated ATP hydrolysis. Panels A, B, C, and D illustrate the effects of digoxin, digitoxin, digoxigenin, and digitoxigenin on Pgp-mediated ATP hydrolysis, respectively. The ATPase activity in the presence of increasing concentrations of all test compounds exhibited a monophasic curve, indicating a single binding site for these drugs on Pgp. The basal activity of Pgp in the absence of drugs (dotted lines) was consistent with previously observed data, averaging 380.58 ± 90.41 nmol min^−1^ mg^−1^ (Figure 2) [36,44]. The kinetics were fit considering the basal activity using a modified Michaelis-Menten equation (Equation (1)). The resulting values were maximum velocity (*V_max_*), which denotes the maximum rate of activity at a saturated drug concentration, and the Michaelis-Menten constant (*K_m_*), which represents the substrate concentration required to allow an enzyme to function at half its maximum velocity. The extracted *V_max_* values from fitting the kinetic curves to Equation (1) were relative to the offset (basal activity) in each experiment. The estimated *V_max_* and *K_m_* values for digoxin (Figure 2A) from kinetic fitting were 277.53 ± 7.24 nmol min^−1^ mg^−1^ and 85.63 ± 8.38 µM, respectively. The *K_m_* value for digoxin aligned with the previously reported value observed in human Pgp-enriched insect cell membranes [45]. For digitoxin (Figure 2B), the *V_max_* and *K_m_* values were 50.7 ± 13.1 nmol min^−1^ mg^−1^ and 99.19 ± 13.3 µM, respectively. Previous studies have reported that some Pgp substrates reduce Pgp-mediated ATPase activity to below basal levels [2,46,47]. Although the mechanism of digitoxin-induced decrease in Pgp-mediated ATPase activity is not well understood, a possible theory that can explain this phenomenon is based on previous studies that have reported elevated basal levels of Pgp-mediated ATPase activity in the presence of endogenous substrates, such as cholesterol in the lipid bilayer [48]. However, these endogenous substrates are not transported by Pgp [49], but they can be flip-flopped between the leaflets of the plasma membrane bilayer [50,51]. Thus, a drug interacting at similar binding sites as the endogenous substrates but at a slower rate can appear to inhibit the observed rate of ATPase activity since the Pgp-mediated ATPase activity accounts for both basal and drug-induced activation. In the case of digoxigenin (Figure 2C), the *V_max_* and *K_m_* values were 541.92 ± 111 nmol min^−1^ mg^−1^ and 426.82 ± 173.26 µM, respectively. The kinetic fitting for digitoxigenin (Figure 2D) showed *V_max_* and *K_m_* values of 216.02 ± 16.01 nmol min^−1^ mg^−1^ and 67.58 ± 19.76 µM, respectively. The kinetic data for CG-induced Pgp-mediated ATP hydrolysis are summarized in Table S1.

### 2.2. Quenching of Pgp’s Intrinsic Tryptophan Fluorescence to Determine Drug-Protein Binding Affinity

**Figure 3:**
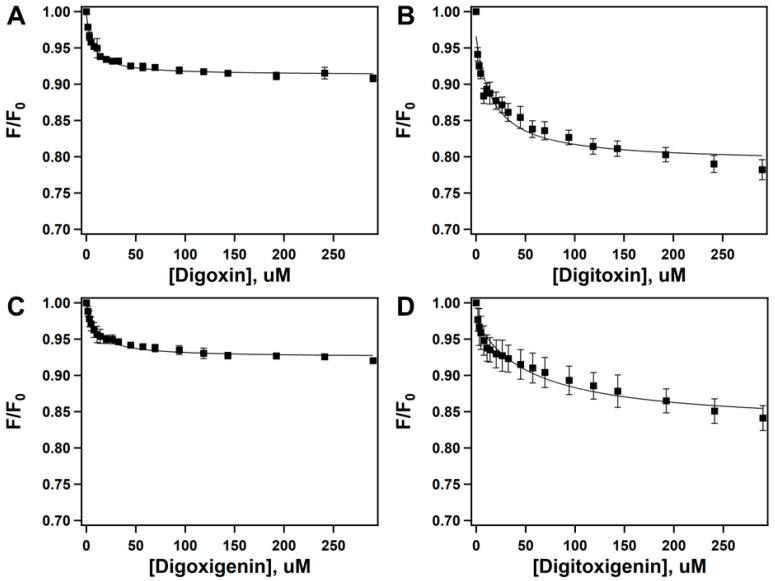
The drug-protein binding affinity of CGs to Pgp was determined through the intrinsic fluorescence of tryptophan residues in Pgp. The drug-induced fluorescence of Pgp at 330 nm in the presence of (**A**) digoxin (closed squares), (**B**) digitoxin (closed squares), (**C**) digoxigenin (closed squares), and (**D**) digitoxigenin (closed squares). The solid line in each panel represents the corrected fluorescence (*F_corrected_*) as a function of varying concentration of the CGs. Each data point represents the average of at least three replicates. Error bars indicate the standard deviation.

Previous studies have indicated the use of a spectrophotometric approach to measure changes in the intrinsic tryptophan fluorescence of proteins induced by drugs as a robust tool for determining drug-protein binding affinities [52,53]. Figure 3 shows the effect of increasing concentrations of CGs on the fluorescence of Pgp at 330 nm upon excitation at 295 nm. The corrected fluorescence (*F_corrected_*) was plotted against the presence of an increasing concentration of digoxin (Figure 3A), digitoxin (Figure 3B), digoxigenin (Figure 3C), and digitoxigenin (Figure 3D).

The fluorescence kinetics were fitted to Equation (3). All four CGs tested showed a monophasic trend, suggesting a single binding site for each drug. The Stern-Volmer constant (*K_sv_*) values from CG-induced tryptophan fluorescence in Pgp for digoxin, digitoxin, digoxigenin, and digitoxigenin were 0.14597 ± 0.0117 µM^−1^, 0.059841 ± 0.0159 µM^−1^, 0.09839 ± 0.0146 µM^−1^, and 0.01765 ± 0.0048 µM^−1^, respectively. The *K_sv_* values for the tested CGs decreased significantly when tested at a higher temperature (37 °C), indicating that, as expected, CG-induced quenching of Pgp’s intrinsic fluorescence happens predominantly via static, rather than dynamic, quenching. Static quenching is known as fluorescence quenching due to drug-protein complex formation, whereas fluorescence quenching caused by random collisions between the drug and the protein is known as dynamic quenching [36]. Previous studies have demonstrated that if the quenching is static, the *K_sv_* value correlates with the drug–protein binding affinity, and the resulting value is the dissociation constant (*K_D_*), which is the substrate concentration required to keep the enzyme half-saturated [3,36]. The *K_D_* values recorded from drug-induced Pgp fluorescence for digoxin, digitoxin, digoxigenin, and digitoxigenin were 6.85 ± 0.83 µM, 16.71 ± 4.44 µM, 10.16 ± 0.01 µM, and 56.66 ± 15.55 µM, respectively. The observed dissociation constants (*K_D_*) of CGs in the order of how tightly they bound to Pgp were digoxin > digoxigenin > digitoxin > digitoxigenin. The tryptophan quenching studies in this study were performed in the absence of ATP and/or co-factors. Previous studies have suggested that the addition of various nucleotides in the presence of drug substrates had only a small effect on the solvent accessibility of the tryptophan residues in Pgp [54]. Similar results were observed during our initial studies. A summary of the Stern-Volmer constants (*K_sv_*) and dissociation constants (*K_D_*) for the CG–Pgp binding affinity curves is shown in Table S2.

### 2.3. Acrylamide Quenching of Protein Fluorescence to Determine CG-Induced Conformational Changes in Pgp

The tertiary conformation of Pgp can be altered upon its interaction with a drug [55,56], resulting in a change in the microenvironment of the protein’s intrinsic tryptophan residues that affects the solvent-accessibility of such residues. This phenomenon can be utilized to indirectly probe Pgp’s conformation. Acrylamide, an aqueous, polar collisional quencher, can be used to determine changes in the solvent-accessibility of Pgp’s intrinsic tryptophan residues measured with the help of spectrophotometric analysis [57,58]. Acrylamide quenching of intrinsic tryptophan residues in Pgp was used to analyze the drug-induced conformational changes in Pgp when interacting with digoxin, digitoxin, digoxigenin, and digitoxigenin.

Figure S1 shows the Stern-Volmer plots of CG-induced fluorescence quenching of the intrinsic tryptophan residues in Pgp by acrylamide in the presence of 250 µM of the drugs (solid lines). The resulting value was a Stern-Volmer constant (*K_sv_*), which represents the availability of the quencher to the excited fluorophore. The fluorescence kinetics of the Stern-Volmer plot (*F_0(corrected)_/F_(corrected)_*) versus acrylamide concentration were fitted to Equation 4. The Stern-Volmer plot of Pgp in the absence of drug, apo-Pgp (dashed line), and Pgp in the presence of 3.2 mM AMPPNP (dotted line), a non-hydrolysable form of ATP (Pgp + AMPPNP), respectively, is represented in Figure S1. apo-Pgp has an inward-open conformation, and its *K_sv_* value was 2.5 ± 0.04 M^−1^, which was consistent with previous studies [54]. The *K_sv_* value of NATA, a free analog of tryptophan, was 19.3 ± 0.18 M^−1^ (dot-dashed line), as previously reported (Figure S1) [2,54]. The *K_sv_* value of NATA was approximately tenfold higher than that of apo-Pgp, suggesting that not all tryptophan residues were available for quenching and also indicating that most of Pgp’s tryptophan residues were in a hydrophobic environment [54]. The *K_sv_* value of Pgp in the presence of a non-hydrolysable form of ATP, AMPPNP, was 1.6 ± 0.01 M^−1^, which was comparable to previously observed data [1] (Figure S1). Cryo-electron microscopy and X-ray structures of Pgp in the presence of AMPPNP have revealed that Pgp exhibits an outward-open conformation [59,60,61].

**Figure 4:**
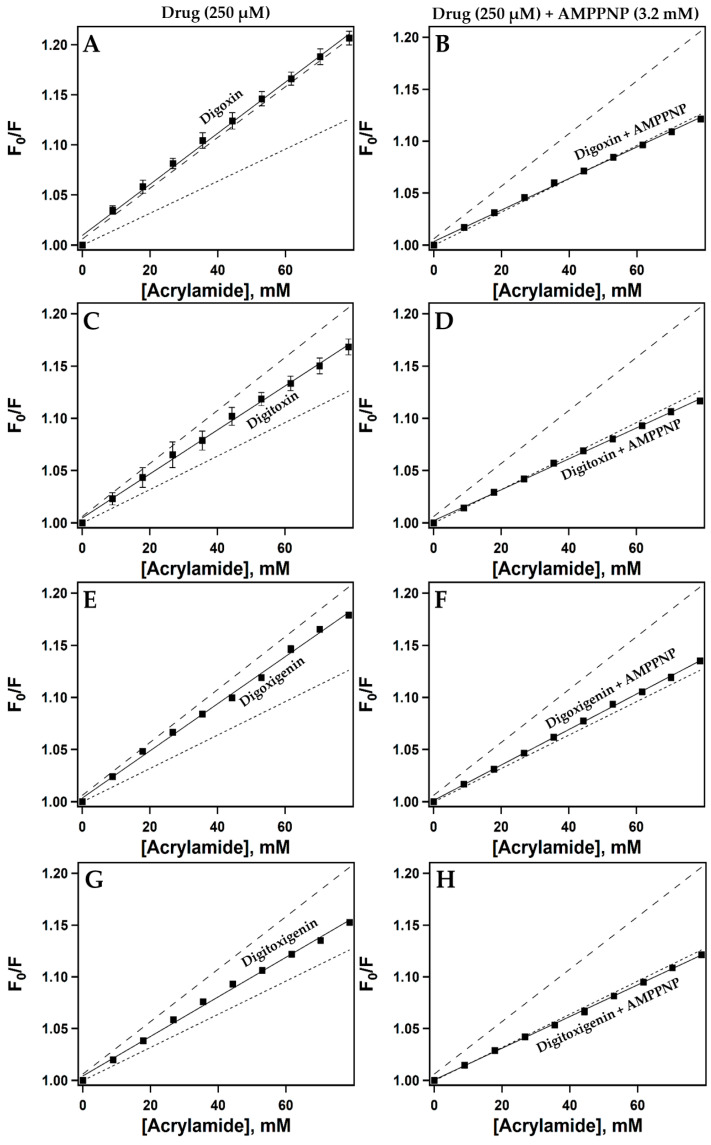
CG-induced conformational changes in Pgp determined by acrylamide as an aqueous quencher. Stern-Volmer plots of Pgp induced by CGs. The solid lines in panels (**A**,**C**,**E**,**G**) represent the Stern-Volmer plots of Pgp induced by digoxin, digitoxin, digoxigenin, and digitoxigenin in the absence of AMPPNP, respectively. The solid lines in panels (**B**,**D**,**F**,**H**) represent the Stern-Volmer plots of Pgp induced by digoxin, digitoxin, digoxigenin, and digitoxigenin in the presence of 3.2 mM AMPPNP, respectively. The dashed line and dotted line in each panel represent the Stern-Volmer plots of Apo-Pgp and Pgp + 3.2 mM AMPPNP, respectively. Each data point represents the average of at least three replicates. Error bars indicate the standard deviation.

In each panel of Figure 4, the Stern-Volmer plot for apo-Pgp (dashed line) and Pgp in the presence of 3.2 mM AMPPNP (dotted line) is depicted for comparison. The solid lines in Figure 4A,C,E,G show the drug-induced *K_sv_* values of Pgp with digoxin, digitoxin, digoxigenin, and digitoxigenin in the absence of 3.2 mM AMPPNP, respectively. The drug-induced *K_sv_* values of Pgp with digoxin, digitoxin, digoxigenin, and digitoxigenin in the presence of saturating concentrations of 3.2 mM AMPPNP are represented by solid lines in Figure 4B,D,F,H, respectively. The digoxin-induced *K_sv_* values of Pgp in the absence and presence of AMPPNP were 2.55 ± 0.06 M^−1^ and 1.51 ± 0.02 M^−1^, respectively. The *K_sv_* values of Pgp induced by digitoxin in the absence and presence of a saturating concentration of AMPPNP were 2.10 ± 0.04 M^−1^ and 1.48 ± 0.02 M^−1^, respectively. The digoxigenin-induced *K_sv_* values of Pgp in the absence and presence of AMPPNP were 2.26 ± 0.04 M^−1^ and 1.70 ± 0.01 M^−1^, respectively. The drug-induced *K_sv_* values of Pgp with digitoxigenin in the absence and presence of AMPPNP were 1.91 ± 0.04 M^−1^ and 1.51 ± 0.02 M^−1^, respectively. All four drugs tested in this study had a significant effect on the *K_sv_* value of Pgp in the presence of a saturating concentration (3.2 mM) of AMPPNP, suggesting a conformational change in the tertiary structure of Pgp that interacted with the drugs, shifting toward an outward-open conformation. A summary of the Stern-Volmer constants (*K_sv_*) and their standard deviations for CG-induced conformational changes in the intrinsic fluorescence of Pgp is shown in Table S3.

### 2.4. Interaction of Cardiac Glycosides with Pgp Determined by STDD NMR

**Figure 5:**
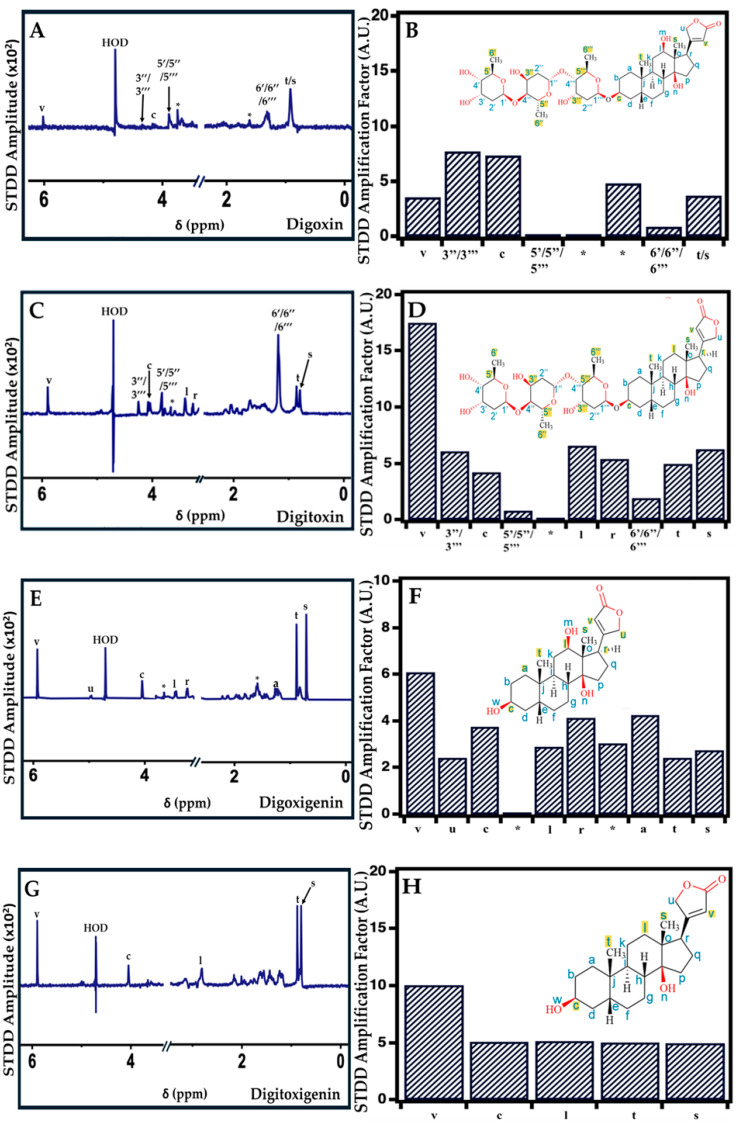
STDD NMR spectra of digoxin, digitoxin, digoxigenin, and digitoxigenin with 1 µM Pgp. The STDD NMR spectra of (**A**) digoxin, (**C**) digitoxin, (**E**) digoxigenin, and (**G**) digitoxigenin are shown (blue). The amplification factors of (**B**) digoxin, (**D**) digitoxin, (**F**) digoxigenin, and (**H**) digitoxigenin are also shown. The protons on the drug that interact with Pgp are also indicated in each of the drug structures (highlighted in yellow). In this study, the concentration of the drug used was 1 mM. HOD (Partially deuterated water). The ‘*’ denotes unidentified peaks.

Previously, STDD NMR has been used as an effective tool to identify the interactions between the functional groups of a ligand and protein [62,63]. In this study, STDD NMR was used to probe the specific moieties on the drugs involved in the CG–Pgp interaction. Figure 5 shows the STDD NMR spectra for the CG–Pgp interaction with 1 mM (A) digoxin, (C) digitoxin, (E) digoxigenin, and (G) digitoxigenin. The amplification factors for (B) digoxin, (D) digitoxin, (F) digoxigenin, and (H) digitoxigenin, along with their highlighted parts in the structures of the drug involved in the interaction with 1 µM Pgp for comparison (red circles), are also shown. For digoxin (Figure 5B), the saturation transfer was prominent in protons associated with carbons (C3′′, 3′′′, 5′, 5′′, 5′′′, 6′, 6′′, and 6′′′) on the sugar groups. Digoxin also displayed an interaction with Pgp in protons associated with C (c and t/s) on the steroid core and C (v) on the lactone ring of the drug. The STDD NMR results for the digoxin–Pgp interaction were relatively comparable to those previously published [36]. In the case of digitoxin (Figure 5D), the STDD NMR signals were most significant in protons associated with C (3′′, 3′′′, 5′, 5′′, 5′′′, 6′, 6′′, and 6′′′) on the sugar groups. The digitoxin–Pgp interaction was also observed in protons associated with C (l, r, t, and s) in the steroid core and protons associated with C (v) on the lactone ring. Based on our results, digoxigenin (Figure 5F) exhibited an interaction with Pgp in protons associated with C (c, l, r, a, t, and s) in the steroid core. The digoxigenin–Pgp interaction was most prominent in protons associated with C (v and u) in the lactone ring. Digitoxigenin (Figure 5H) displayed a prominent interaction in protons associated with the lactone ring, C (v), and also showed an interaction in protons associated with C (c, l, t, and s) in the steroid core. Overall, the CG–Pgp interaction was primarily observed in the sugar groups of the glycones digoxin and digitoxin, as well as in the steroid core and lactone ring. Digitoxin showed the highest interaction in the lactone ring, whereas the aglycones digoxigenin and digitoxigenin displayed interactions mainly in the steroid core and less interaction in the lactone ring.

## 3. Discussion

P-glycoprotein (Pgp) plays a pivotal role in the disposition of cardiovascular drugs by modulating their bioavailability, which can often result in adverse drug reactions (ADRs), decreased drug efficacy, and drug toxicity [8,10,36]. To overcome Pgp-mediated effects on drugs, several Pgp inhibitors have been identified, with little to no success, proving to be highly challenging [64,65]. Moreover, it does not help the cause that most Pgp substrates are lipophilic, which presents additional challenges when these drugs are subjected to experimental analysis [50].

In this study, a comparative analysis of the effects of several cardiac glycosides (CGs) on the ATPase activity of Pgp, the CG–Pgp binding affinities, and CG-induced conformational changes in the tertiary structure of Pgp was performed. Furthermore, we employed saturation transfer double-difference (STDD) NMR to investigate the functional groups in CGs responsible for their interaction with Pgp. Additionally, Pgp-mediated transport data, accumulation assays, and competitive studies are available in the published literature and were utilized to facilitate a comparison with our data and subsequently propose a model for the CG–Pgp interaction.

**Figure 6:**
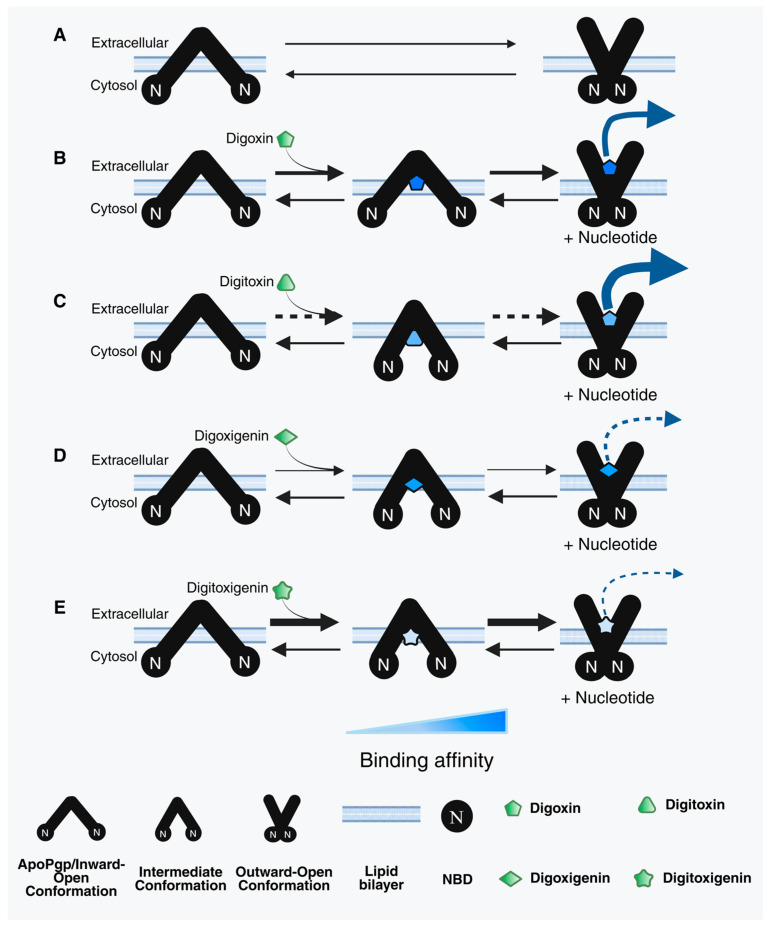
Proposed model for cardiac glycoside (CG)-induced Pgp-mediated ATP hydrolysis and transport. The three conformations of Pgp shown in the graphical representation are inward-open, drug-bound intermediate, and outward-open conformations, respectively. The panel shows (**A**) Apo-Pgp and Pgp-mediated transport of (**B**) digoxin, (**C**) digitoxin, (**D**) digoxigenin, and (**E**) digitoxigenin. N represents the nucleotide-binding domain (NBD). The intensity of blue color in the drug represents the binding affinity. The intensity of the blue-colored arrow represents the rate of transport (solid/thicker arrow = higher transport liability; dotted/narrower arrow = lower transport liability).

Figure 6 illustrates the proposed models for the relationships between Pgp and each of the CGs in this study, coupled with the transport data shown in the literature. Panel A displays the two primary conformations of Pgp for reference, inward open and outward open, representing the structural assembly of Pgp. The inward-open conformation is displayed when the cytosolic side of Pgp is exposed to the bulk solvent, and the nucleotide-binding domains (NBDs) are exposed far apart from each other. The inward-open orientation can be associated with Pgp’s unbound state. The outward-open conformation of Pgp suggests that the extracellular sides are exposed to the bulk solvent and is associated with its effluxing orientation. Many substrates, upon binding to Pgp, are shown to induce an “intermediate” conformation of Pgp, in between its inward-open and outward-open states, when both the cytosolic and extracellular sides are equally exposed to the bulk solvent [3,36].

Our results suggest that Pgp’s ATPase hydrolytic activity is induced by digoxin (Figure 2A) in a monophasic fashion, with observed *V_max_* and *K_m_* values of 277.53 ± 7.24 nmol min^−1^ mg^−1^ and 85.63 ± 8.38 µM, respectively. Digitoxin, a glycone-like digoxin without the hydroxyl group on the C12 position [66], also displayed a monophasic trend but reduced Pgp’s ATPase hydrolytic activity below basal levels. The observed *V_max_* and *K_m_* values of digitoxin were 50.7 ± 13.1 nmol min^−1^ mg^−1^ and 99.19 ± 13.3 µM, respectively (Figure 2B). While this may seem surprising, considering their almost identical structures, a previous study reported that the addition of a singular hydroxyl group in meperidine analogs significantly increased the Pgp–substrate activity [67]. Perhaps this may explain the differences observed in Pgp’s ATPase hydrolytic activity in the presence of digoxin or digitoxin. In this study, we also observed that digoxin’s dissociation constant (*K_D_*) from Pgp was 6.85 ± 0.83 µM compared to that of digitoxin (*K_D_* = 16.71 ± 4.44 µM) (Figure 3A,B), suggesting that digitoxin more readily dissociates from Pgp than digoxin. Although digitoxin’s dissociation constant was higher (indicating lower binding affinity) than that of digoxin, our indirect conformational analysis suggests that digitoxin alters the tertiary structure of Pgp to an “intermediate” conformation (*K_sv_* value of 2.10 ± 0.04 M^−1^) in the absence of a nucleotide (Figure 4C). By contrast, digoxin appears to have no effect on Pgp’s conformation upon binding (Figure 4A), with an observed *K_sv_* value of 2.55 ± 0.06 M^−1^. Upon the addition of a nucleotide, Pgp’s conformation shifted to “outward open” in the presence of both digoxin and digitoxin (Figure 4B,D), with *K_sv_* values of 1.51 ± 0.02 M^−1^ and 1.48 ± 0.02 M^−1^, respectively. It is possible that the distance between Pgp’s NBDs may influence the rate of Pgp-mediated transport, and compounds that even partially breach this barrier by moving the NBDs closer in proximity may be translocated by Pgp faster than those that do not [2,68]. A previous study reported that the transepithelial transport rate of digitoxin in porcine kidney epithelial cells stably expressing human MDR1 cDNA, L-MDR1 cells, was approximately 2.5-fold higher than that of digoxin [31]. Considering that digitoxin dissociates from Pgp more readily and shifts Pgp to an “intermediate” conformation in the absence of a nucleotide, this could possibly explain the higher efflux liability of digitoxin as compared to that of digoxin.

The aglycones digoxigenin and digitoxigenin induced Pgp’s ATPase hydrolysis activity (Figure 2C,D) and displayed monophasic trends. The observed *V_max_* and *K_m_* values for digoxigenin-induced effect on Pgp’s ATPase activity were 541.92 ± 111 nmol min^−1^ mg^−1^ and 426.82 ± 173.26 µM, respectively. However, digitoxigenin-induced Pgp’s ATPase activity showed *V_max_* and *K_m_* values of 216.02 ± 16.01 nmol min^−1^ mg^−1^ and 67.58 ± 19.76 µM, respectively. Our data for Pgp’s ATPase activity in the presence of aglycones suggests that digitoxigenin modulates Pgp’s activity faster than digoxigenin. We also observed that the binding affinity of the digoxigenin–Pgp complex (*K_D_* = 10.16 ± 0.01 µM) was significantly higher than that of digitoxigenin’s dissociation constant from Pgp (*K_D_* = 56.66 ± 15.55 µM) (Figure 3C,D), suggesting that digitoxigenin has a weaker binding and dissociates more readily from Pgp than digoxigenin. Both the aglycones used in this study, digoxigenin (*K_sv_* = 2.26 ± 0.04 M^−1^) and digitoxigenin (*K_sv_* = 1.91 ± 0.04 M^−1^), upon binding to Pgp, shifted it to an “intermediate” conformation in the absence of a nucleotide (Figure 4E,G). In the presence of a nucleotide, both digoxigenin (*K_sv_* = 1.70 ± 0.01 M^−1^) and digitoxigenin (*K_sv_* = 1.51 ± 0.01 M^−1^) altered the tertiary conformation of Pgp to outward open (Figure 4F,H). While transport studies of aglycones are relatively sparse, especially for digitoxigenin, competitive studies have demonstrated the minimal effect of the two aglycones on the transport of [(3)H]-N-methyl-quinidine [NMQ] by Pgp [32]. On the other hand, glycones are seen to significantly reduce Pgp-mediated NMQ transport. Considering that all four of these compounds share the same core structure, one possibility that can be hypothesized from these data is that aglycones interact weakly, if at all, with Pgp.

The magnitudes of the STDD amplitudes derived from the 1D H^1^ STDD NMR spectra displayed interactions that were prominent in protons associated with C (v, t, and s) in all four drugs when interacting with Pgp (Figure 5). The glycones digoxin (Figure 5B) and digitoxin (Figure 5D) also showed interactions with Pgp in the protons associated with carbon atoms in the sugar group, with digitoxin’s interaction appearing to be of a higher degree. A previous study reported that the δ-lactone ring and the tri-D-digitoxose sugar moieties attached to the 3β-position of the steroid core in cardiac glycosides influence their interaction with Pgp [32]. Overall, the transport rates of digoxin and digitoxin in the literature appear to be similar or slightly higher for digitoxin [31], despite digitoxin’s reduction of Pgp’s ATPase activity. This can potentially be explained by the interaction between digitoxin and Pgp displayed by STDD NMR. Digitoxin appears to interact with Pgp at more proton locations than digoxin, and with overall slightly higher amplification factors. In the case of digoxigenin, its significantly higher *K_m_* value and lack of sugar groups likely explain its lower efflux liability as compared to digoxin and digitoxin. The apparent lowest efflux liability of digitoxigenin is likely due to its significantly lower binding affinity compared to the other CGs, paired with its significantly less interaction.

The efflux protein Pgp plays a pivotal role in transporting not only several drugs but also other toxic substances from cells; therefore, complete inhibition of Pgp may do more harm than good to sensitive tissues and organs by exposing them to vulnerable positions. Inhibiting Pgp using drugs such as zosuquidar [69], PSC-833 [70], and cyclosporine [71] has been long studied, including several clinical trials, as a mechanism to effectively treat several diseases, such as acute myeloid leukemia (AML) [72]. The initial studies showed promising results; however, the outcomes of the clinical trials did not demonstrate a significant benefit in preventing remission or improving survival rates in patients who were not treated with Pgp inhibitors [69]. In general, the clinical studies also provided evidence contrary to the concept that ATP cassette transporter inhibition can improve the survival rate of patients. The study also adds significant value, allowing future researchers to redirect their resources toward redesigning existing drugs or synthesizing next-generation drugs rather than spending valuable resources on studies targeting the inhibition of Pgp.

**Figure 7:**
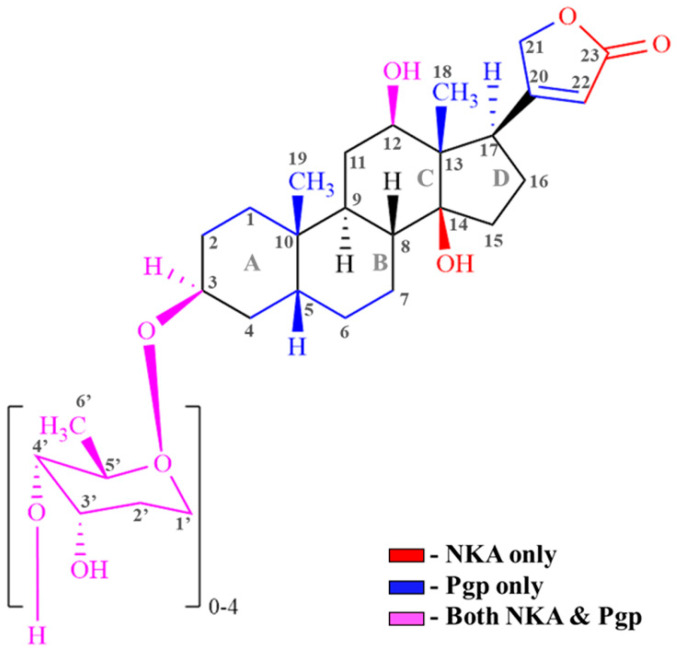
The general structure of cardiac glycosides (CGs) shows the interactions of its specific regions/groups with Na^+^/K^+^ ATPase (NKA) (red), Pgp (blue), and both NKA and Pgp (pink).

Table 1 demonstrates the interactions of CGs with Na^+^/K^+^ ATPase (NKA) and Pgp. Fitting the data generated in Table 1 into the general structure of CGs (Figure 7) paves the way for designing next-generation CGs. Our data suggest that the functional groups in the CG steroid core, such as replacing the methyl group (-CH3) with a hydroxyl (-OH) or a trifluoromethyl (CF3) group, can be potential targets and offer flexibility for designing next-generation CGs that would retain their NKA activity while reducing their affinity for Pgp.

## 4. Materials and Methods

### 4.1. Chemical Reagents

Digoxin was purchased from Alfa Aesar (Tewksbury, MA, USA). Digitoxin was obtained from TCI America (Portland, OR, USA). (3β,5β)-3,14-Dihydroxy-card-20(22)-enolide (digitoxigenin) was purchased from Cayman Chemical Co. Inc. (Ann Arbor, MI, USA). 3β,12β,14-Trihydroxy-5β,20(22)-cardenolide (digoxigenin) was acquired from Astra Tech (Bristol, PA, USA). Adenosine 5′-(β,γ-imido) triphosphate lithium salt (AMPPNP), ammonium chloride (NH4Cl), 4-(2-hydroxyethyl1)-1-piperazine ethane sulfonic acid (HEPES), N-acetyl-L-tryptophanamide (NATA), and acrylamide were purchased from Sigma-Aldrich (Milwaukee, WI, USA). Imidazole and ethylene glycol tetra-acetic acid (EGTA) were secured from Alfa Aesar (Tewksbury, MA, USA). Sodium orthovanadate (Na3VO4) was obtained from Enzo Life Sciences (Farmingdale, Long Island, NY, USA). Nitrilotriacetic acid (NTA) resin was purchased from Thermo Fisher Scientific (Waltham, MA, USA). Dithiothreitol (DTT) was obtained from Gold Biotechnology (Olivette, MO, USA). Disodium ATP, Tris-HCl, and cholesterol were acquired from Amresco (Solon, OH, USA). The detergent, n-dodecyl-β-D-maltoside (DDM), was purchased from EMD Millipore Corporation (San Diego, CA, USA). Deuterium oxide 99.9 atom% D and dimethyl sulfoxide-d6 99.9 atom% D were acquired from Sigma-Aldrich, Burlington, MA, USA. Escherichia (*E. coli*) total lipid extract powder was obtained from Avanti Polar Lipids Inc. (Alabaster, AL, USA). All other chemicals and reagents used in this study, not mentioned above, were purchased from Thermo Fisher Scientific, unless otherwise declared.

### 4.2. Expression and Purification of P-Glycoprotein (Pgp)

Overexpression of wild-type mouse Pgp (MDR3) was achieved by using genetically engineered strains of Pichia pastoris with a 6X histidine tag. The cells were cultured in glycerol media, and methanol was used to induce the overexpression of Pgp [78,79] at the Bioexpression and Fermentation Facility (BFF), University of Georgia, Athens, GA, USA. A repeated cycle of freezing using liquid nitrogen, blending, and thawing was used to lyse the Pgp-expressing *P. pastoris* cells [80]. The protein was purified using affinity chromatography with nickel-nitrilotriacetic acid (Ni-NTA) resin (Thermo Fisher Scientific, Waltham, MA, USA) [79]. DDM was used to solubilize the protein, and the solution was concentrated using Amicon Ultra-15 100 kDa cut-off filters (EMD Millipore, Billerica, MA, USA). The concentration of purified protein was measured at 280 nm using a calculated extinction coefficient of 1.28 per mg mL^−1^ on a Nanodrop (DeNovix, Wilmington, DE, USA) [81]. The purity of the protein was determined to be over 95% using SDS-PAGE analysis [79]. The purified DDM-solubilized Pgp was aliquoted and stored at −80 °C for future analysis.

### 4.3. Preparation of Proteoliposomes

Pgp was reconstituted into liposomes as previously described [1]. Briefly, liposomes comprising 80% (*w*/*v*) Avanti Escherichia coli total lipid extract and 20% (*w*/*v*) cholesterol were dissolved in chloroform to achieve a final concentration of 10 mg mL^−1^, and the volume was adjusted to 10 mL. The resulting organic solution was vacuum-dried by evaporation using a Buchi rotavapor, model R-114, and then resuspended in 10 mL of rehydration buffer (0.1 mM EGTA and 50 mM Tris-HCl solution, pH 7.4). The solution was subjected to ten cycles of freeze–thaw using liquid nitrogen and a water bath (67 °C), resulting in the formation of liposomes with varying sizes, and extruded eleven times in a LIPEX extruder (Northern Lipids, Burnaby, BC, Canada) to produce identical-sized liposomes, using a 400 nm cut-off filter.

Approximately 40 µL of DDM-solubilized Pgp was dialyzed for two hours against HEPES buffer (20 mM HEPES, 100 mM NaCl, 5 mM MgCl_2_, 2 mM DTT, pH 7.4) to remove any remnants of the detergent, DDM. The dialyzed protein was mixed with 4 mg mL^−1^ of the extruded liposomes and incubated on ice for at least one hour to allow Pgp to integrate into the liposomes. To remove any additional detergent and further facilitate Pgp integration into the liposome, the solution was dialyzed for a second time for two hours in HEPES buffer to form proteoliposomes, and the concentration of Pgp reconstituted in liposomes was determined using the Bio-Rad DC Protein assay. Finally, the solution was aliquoted and stored at −80 °C for future use.

### 4.4. Measurement of Pgp-Mediated ATPase Activity

Pgp-mediated ATP hydrolysis in the presence of digoxin, digitoxin, digoxigenin, and digitoxigenin was measured as previously described using Chifflet’s procedure [82]. The Chifflet method is a colorimetric method that measures the amount of free inorganic phosphate (Pi) through the formation of ammonium molybdate and Pi complex, which produces a strong absorbance at 850 nm. The absorbance was measured on a 96-well plate using a CLARIOstar microplate reader (BMG Labtech, Ortenberg, Germany). To measure the drug-induced ATPase activity of Pgp, Chifflet buffer (150 mM NH_4_Cl, 5 mM MgSO_4_, 0.02% *w*/*v* NaN_3_, 50 mM Tris-HCl, pH 7.4), 250 nM proteoliposome, and varying concentrations of digoxin, digitoxin, digoxigenin, and digitoxigenin were incubated at 37 °C. The final concentrations of the proteoliposome and ATP were maintained at 50 nM and 3.2 mM, respectively. Orthovanadate was used as a control and run in parallel to the samples. Finally, the kinetic curves for ATPase activity were fit to a modified Michaelis-Menten equation (Equation (1)) [83]. Igor 6.2 Pro software (Wavemetrics, Tigard, OR, USA) was used for data analysis.

(1) $$V=\frac{V_{\max\left[S\right]}}{K_{m}+\left[S\right]}+V_{basal}$$

where *V* = Rate of ATP hydrolysis; *V_max_* = Maximum velocity of ATP hydrolysis; [*S*] = Substrate concentration; *K_m_* = Michaelis–Menten constant; and *V_basal_* = basal ATPase activity.

### 4.5. Pgp-Drug Binding Affinity by Intrinsic Tryptophan Quenching

The intrinsic tryptophan residues in the protein can be selectively excited and emitted at 295 nm and 330 nm wavelengths, respectively. The binding of the drug to the protein can alter the spatial arrangement encountered by the tryptophan residue(s) and thereby regulate the fluorescence intensity of the protein [52]. Previous studies have utilized intrinsic tryptophan quenching to determine the binding affinity of drugs to Pgp [54,57,84]. Proteoliposomes diluted to 1 µM in Chifflet buffer containing 2 mM DTT were titrated against varying concentrations of drugs. The intrinsic protein fluorescence was measured at 330 nm upon excitation at 295 nm using an Olis DM 45 spectrophotometer (Olis Corporation, Bogart, GA, USA). To nullify the effect of Rayleigh bands on the observed spectra, a 10 nm band-pass filter was inserted on the excitation and emission paths of the spectrophotometer. The drug-induced quenching of intrinsic Pgp fluorescence was corrected (*F_corrected_*) for factors such as dilution, background fluorescence, and inner filter effects using the equation mentioned below (Equation (2)) [84,85].

(2) $$F_{corrected}=\left(F-B\right)10^{\frac{\left(\varepsilon_{ex}b_{ex}+\varepsilon_{em}b_{em}\right)\left[Q\right]}{2}}$$

where *F_corrected_* = Drug-induced quenching of intrinsic Pgp fluorescence; *F* = Emitted intrinsic Pgp fluorescence at 330 nm; [*Q*] = Concentration of drug; *ε_ex_ and ε_em_* = Excitation and emission extinction coefficients, respectively; *b_ex_ and b_em_* = Path lengths of excitation (0.4 mm) and emission (10 mm) wavelengths, respectively; and *B* = Background fluorescence measured from drug and solvent.

All four drugs, digoxin, digitoxin, digoxigenin, and digitoxigenin, were essentially transparent above 250 nm, and their absorbance did not interfere with the experiments. Fluorescence quenching observed due to complex formation between the drug and protein before excitation is known as static quenching, and quenching observed due to the random collision of the drug with the protein is called dynamic quenching [85]. In this study, all four drugs exhibited static quenching. The resulting quenching curves were fit using a modified Stern-Volmer equation (Equation (3)), as follows:

(3) $$F_{corrected}=\frac{F_{O\left(corrected\right)}}{1+K_{sv}\left[Q\right]}+F_{\left(unquenched\right)}$$

where *F_corrected_* = The corrected fluorescence; *F_0(corrected)_* = Fluorescence recorded in the absence of drug; [*Q*] = Concentration of drug; *K_sv_* = Stern–Volmer constant; and *F_(unquenched)_* = unquenched fluorescence.

### 4.6. Drug-Induced Pgp Conformational Changes by Acrylamide Quenching of Protein Fluorescence

Acrylamide, an aqueous collisional quencher, can be used to determine the changes in conformation upon Pgp-drug interaction. The principle of this technique is that as the drug interacts with the protein, the conformation of the accessible tryptophan residues in the protein also changes, resulting in a shift in fluorescence intensity [54,55]. For this experiment, to measure the fluorescence of Pgp reconstituted in liposomes, in the presence (250 µM) or absence of drug(s), the excitation and emission wavelengths used were 295 nm and 330 nm, respectively. The proteoliposomes were diluted to a final concentration of 1 µM in Chifflet buffer (pH 7.4) containing 2 mM DTT, and it was titrated against an increasing concentration of acrylamide. A concentration of 11 µM N-acetyl-L-tryptophanamide (NATA), an analog of tryptophan, was used as a positive control to determine non-specific quenching [47,54]. A saturated concentration of AMPPNP (3.2 mM), a non-hydrolysable form of ATP, was used to generate an outward-open conformation of Pgp. All the experiments for acrylamide quenching were performed at 21 °C. To correct the fluorescence intensities for background, dilution, and inner filter effects, Equation (3) was used. A Stern–Volmer plot was constructed with *F_0(corrected)_/F_(corrected)_* versus acrylamide concentration. The slope of the curve determined the degree of quenching, and it was related to the Stern–Volmer constant (*K_sv_*) by Equation (4) [85], as follows:

(4) $$\frac{F_{o\left(corrected\right)}}{F_{\left(corrected\right)}}=1+K_{sv}\left[Q\right]$$

where *F_0(corrected)_/F_(corrected)_* = Stern–Volmer plot; *K_sv_* = Stern–Volmer constant; and [*Q*] = acrylamide concentration.

### 4.7. Saturation Transfer Double-Difference (STDD) NMR

The STDD NMR technique is a robust method used to characterize the interaction between a drug and receptor protein [36,86]. This technique utilizes a saturated low-strength radio frequency (RF) to selectively excite the receptor protein. The ^1^H STD NMR signal is observed when saturated RF signals are transferred from the protein to the protons on the bound drug that are at a distance of 5 Å, and the distance between the drug and the receptor protein determines the degree of energy transferred and the strength of the drug-protein interaction, which is inversely proportional [62,63]. Due to the localization of Pgp, most of its substrates are lipophilic, resulting in a probable non-specific interaction between its substrates and liposomes in the observed STD NMR spectrum. Therefore, STD NMR of liposomes in the presence of drugs was performed. The resulting spectrum was subtracted from the ^1^H STD NMR spectrum obtained from the drug in the presence of proteoliposome (Pgp reconstituted into liposome) to achieve a saturation transfer double-difference (STDD) NMR spectrum, which nullified the STD observations of the drugs in the presence of liposomes [87,88].

In this study, STDD NMR was used to identify the functional groups of digoxin, digitoxin, digoxigenin, and digitoxigenin involved in the CG–Pgp interaction. This technique was performed as previously described [54,89]. The sample preparation for this experiment included 1 µM proteoliposome (Pgp reconstituted in liposomes) in 100 mM potassium phosphate buffer (80% D2O, 20% ddH20, pH 7.4) along with 1 mM of CGs (either digoxin, digitoxin, digoxigenin, or digitoxigenin), and the control was the same as the samples except it was only the liposomes (devoid of Pgp) in potassium phosphate buffer with 1 mM drug. The NMR tubes with the sample were centrifuged at 3000× *g* for 5 min. To suppress the background signals caused by water, a tailored gradient pulse sequence known as water suppression by gradient-tailored excitation (WATERGATE) was also included. A train of 50 ms Gaussian-shaped selective pulses was used to selectively excite and saturate Pgp for two seconds, and a relaxation delay of five seconds was also used [62,90]. The STD NMR spectra for both the control and the samples were obtained through phase cycling, where the Pgp irradiation resonance was alternated between 1.5 ppm and 42 ppm on and off resonance, respectively, for 512 scans, creating the ^1^H NMR spectrum [57]. The difference observed in the ^1^H NMR spectra of the control and the samples suggested a direct correlation with the degree of interaction between the functional groups of the CGs and Pgp. Equation (5) was used to calculate the STDD amplification factor [36,62], as follows:

(5) $$STDD\;Amplification\;Factor=\frac{\left[l\right]}{\left[P\right]}\frac{\Delta l}{I_{0}}$$

where [*l*] = concentration of drug/ligand; [*P*] = concentration of Pgp; *I*_0_ = amplitude of ^1^H NMR peaks; and ∆*l* = difference observed in the 1H NMR spectra.

For this experiment, the NMR studies were performed on a Bruker Avance NEO 800 MHz NMR spectrometer with a 1.7 mm TCI H{CN} probe at 25 °C. The STD NMR data generated were analyzed using iNMR software (Nucleomatic, Molfetta, Italy), Igor Pro 6.2, and Mnova 14.2.0 (Mastrelab Research S.L., Santiago de Compostela, Spain).

## Figures and Tables

**Table 1:** The interactions of specific regions/groups in cardiac glycosides (CGs) with Na^+^/K^+^ ATPase (NKA), as published in the literature. The interactions of specific groups in CGs with Pgp, as suggested by our STDD-NMR data, is also shown in the table.

Cardiac Glycosides (CG)	Interaction (Region/Group)	
	NKA [73]	Pgp (STDD-NMR Data)
Digoxin	C3 (Sugar); C12 (-OH); C14 (-OH); C17 (Carbonyl) [74,75,76]	Protons on C3,C18, C19 of steroid; C22 of lactone; C3,C5,C6 of sugar
Digitoxin	C3 (Sugar); C14 (-OH); C17 (Carbonyl) [18,77]	Protons on C3,C17, C18, C19 of steroid; C22 of lactone; C3,C5,C6 of sugar
Digoxigenin	C3 (-OH); C12 (-OH); C14 (-OH) [18,73]	Protons on C1, C3,C12, C17, C18, C19 of steroid; C21, C22 of lactone
Digitoxigenin	C3 (-OH); C14 (-OH) [18]	Protons on C1, C12, C18, C19 of steroid; C22 of lactone

## Data Availability

Data are available upon request.

## Supplementary Materials

The following supporting information can be downloaded at: https://www.mdpi.com/article/10.3390/ijms26167813/s1.
