# Supplementary material for: Elucidating the Complex Structural and Molecular Mechanisms Driving P-Glycoprotein-Mediated Transport of Cardiac Glycosides
Source: Int J Mol Sci. 2025 Aug 13;26(16):7813. doi: 10.3390/ijms26167813 (PMC12386283; doi:10.3390/ijms26167813)
Supplement: Supplementary file 1 [file ijms-26-07813-s001.zip › ijms-3788200-supplementary.pdf]

Supplementary Materials

Cardiac Glycosides	Basal Activity (nmol min^{-1} mg^{-1})	V_{max} (nmol min^{-1} mg^{-1})	K_m (μM)
Digoxin	503.35 ± 8.77	277.53 ± 7.24	85.63 ± 8.38
Digitoxin	392.21 ± 4.42	50.7 ± 13.1	99.19 ± 13.3
Digoxigenin	301.532 ± 1.91	541.92 ± 111	426.82 ± 173.26
Digitoxigenin	325.201 ± 23.01	216.02 ± 16.01	67.58 ± 19.76

Table S1. The kinetic parameters and corresponding standard deviations for CG-induced Pgp-mediated ATP hydrolysis.

Cardiac Glycosides	K_{sv} (μM^{-1})	K_D (μM)
Digoxin	0.14597 ± 0.0117	6.85 ± 0.83
Digitoxin	0.059841 ± 0.0159	16.71 ± 4.44
Digoxigenin	0.09839 ± 0.0146	10.16 ± 0.01
Digitoxigenin	0.01765 ± 0.0048	56.66 ± 15.55

Table S2. The Stern-Volmer constants (Ksv) and dissociation constants (KD) with corresponding standard deviations for CG-Pgp binding affinity curves.

Cardiac Glycosides	K_{sv} (M^{-1}) (250 μM drug)	
	**AMPPNP (-)**	**AMPPNP (+)**
Digoxin	2.55 ± 0.06	1.51 ± 0.02
Digitoxin	2.10 ± 0.04	1.48 ± 0.02
Digoxigenin	2.26 ± 0.04	1.70 ± 0.01
Digitoxigenin	1.91 ± 0.04	1.51 ± 0.02

Table S3. The Stern-Volmer constants (Ksv) in the absence or presence of a saturated concentration (3.2 mM) AMPPNP, with corresponding standard deviations for CG-induced conformational changes in the intrinsic fluorescence of Pgp.

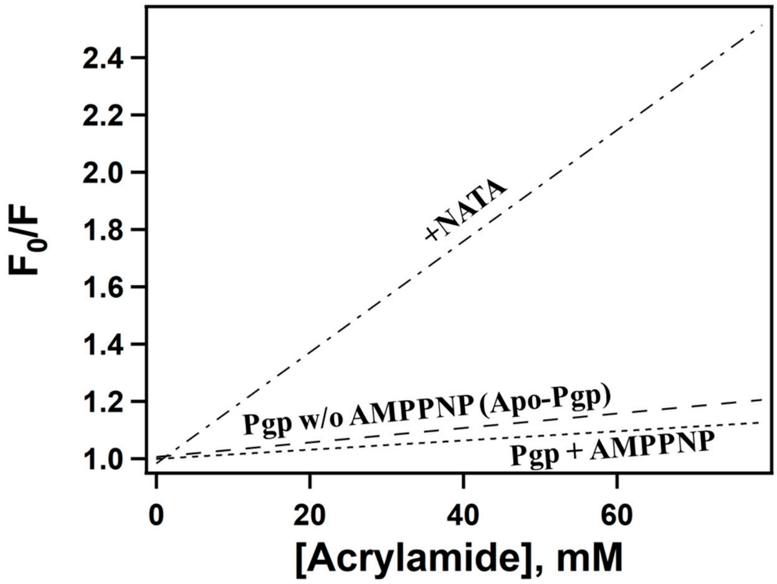

Figure S1. The Stern-Volmer plot of 11 μM NATA (dot-dashed line), Apo-Pgp (dashed line), and Pgp in the presence of 3.2 mM AMPPNP (dotted line).
